# Genetic Variants Affecting Anti-VEGF Drug Response in Polypoidal Choroidal Vasculopathy Patients: A Systematic Review and Meta-Analysis

**DOI:** 10.3390/genes11111335

**Published:** 2020-11-12

**Authors:** Xando Díaz-Villamarín, David Blánquez-Martínez, Ana Pozo-Agundo, Ana María Pérez-Gutiérrez, José Ignacio Muñoz-Ávila, Alba Antúnez-Rodríguez, Ana Estefanía Fernández-Gómez, Paloma García-Navas, Luis Javier Martínez-González, Cristina Lucía Dávila-Fajardo

**Affiliations:** 1 Pharmacy Department, Hospital Universitario Clínico San Cecilio—Instituto de Investigación Biosanitaria (ibs.Granada), 18016 Granada, Spain; efernandez@fibaosalud.com (A.E.F.-G.); palomichi_96@hotmail.com (P.G.-N.); cristinal.davila.sspa@juntadeandalucia.es (C.L.D.-F.); 2 Pfizer-University of Granada-Junta de Andalucía Centre for Genomics and Oncological Research (GENYO), 18016 Granada, Spain; ana.pozo@genyo.es (A.P.-A.); alba.antunez@genyo.es (A.A.-R.); luisjavier.martinez@genyo.es (L.J.M.-G.); 3 Pharmacy Department, Hospital Universitario de Ceuta, 51003 Ceuta, Spain; david.blanquez.sspa@juntadeandalucia.es; 4 Department of Biochemistry and Molecular Biology II, School of Pharmacy, University of Granada, 18011 Granada, Spain; ampergut@correo.ugr.es; 5 Ophthalmology Department—Hospital Universitario Clínico San Cecilio, 18016 Granada, Spain; josei.munoz.sspa@juntadeandalucia.es

**Keywords:** pharmacogenetics, personalized medicine, SNP, anti-VEGF, polypoidal choroidal vasculopathy

## Abstract

Polypoidal choroidal vasculopathy (PCV) is usually regarded as a subtype of choroidal neovascularization (CNV) that is secondary to age-related macular degeneration (AMD) characterized by choroidal vessel branching, ending in polypoidal lesions. Despite their close association, PCV and neovascular AMD have shown differences, especially regarding patients’ treatment response. Currently, antivascular endothelial growth factor (anti-VEGF) drugs, such as ranibizumab, bevacizumab and aflibercept, have demonstrated their efficacy in CNV patients. However, in PCV, anti-VEGF treatments have shown inconclusive results. Many genetic polymorphisms have been associated with a variable response in exudative/wet AMD patients. Thus, the aim of this study is to explore the genetic variants affecting anti-VEGF drug response in PCV patients. In this regard, we performed a systematic review and meta-analysis. We found four variants (*CFH I62V*, *CFH Y402H*, *ARMS2 A69S,* and *HTRA1-62A/G*) that have been significantly related to response. Among them, the *ARMS2 A69S* variant is assessed in our meta-analysis. In conclusion, in order to implement anti-VEGF pharmacogenetics in clinical routines, further studies should be performed, distinguishing physio-pathogenic circumstances between PCV and exudative AMD and the combined effect on treatment response of different genetic variants.

## 1. Introduction

Choroidal neovascularization (CNV) refers to the pathological emergence of new vessels and choroidal invasion of macrophages, myofibroblasts, fibroblasts, and retina pigment epithelium. These new vessels create a microenvironment that promotes macular invasion [1,2]. This suggests that CNV may be secondary to other ophthalmic pathologies such as age-related macular degeneration (AMD) and high myopia.

Polypoidal choroidal vasculopathy (PCV) is usually regarded as a kind of CNV, secondary to AMD. It is characterized by choroidal vessel branching, ending in polypoidal lesions, and it is a result of abnormal aneurysmal neovascular lesions, recurrent neurosensory retinal detachments, and serosanguineous pigment epithelial detachments. However, this remains controversial [3,4]. Indeed, many authors consider PCV as a subtype of exudative/neovascular AMD (nAMD) and/or a different illness [5,6]. Even regarding genetics, variants in the *CFH*, *ARMS2,* and *HTRA1* genes are known to be associated with AMD susceptibility, although information about PCV is scarce. In this regard, the variant *ARMS2* rs10490924 (A69S) has been related to AMD in several publications; however, the poor association reported suggests the presence of other genes that are especially related to PCV, affecting its pathogenicity.

Despite being associated with nAMD, PCV shows many differences from typical nAMD, and those differences related to treatment response are significantly relevant [4,7,8,9].

Currently, ranibizumab (Lucentis™) and bevacizumab (Avastin™), both antivascular endothelial growth factor (anti-VEGF) recombinant humanized monoclonal antibodies, and aflibercept (Eylea™), a fusion protein that includes extracellular domains of VEGF receptors 1 and 2, have demonstrated their efficacy in CNV, secondary to AMD patients.

However, anti-VEGF treatment has shown inconclusive results in PCV patients, showing a worse response and greater resistance to treatment than nAMD. Among these patients, using intravitreal anti-VEGF drugs have not been associated with a significant improvement of vascular and polypoidal lesions [10,11,12,13,14,15,16,17,18]. On the other hand, they have been demonstrated to improve the typical hemorrhage and exudation that characterize these patients [10,19,20]. On this basis, photodynamic therapy (PDT) is still frequently used in PCV patients, although it is related to severe adverse drug events such as higher recurrent rates and higher risk of complications (e.g., retinal pigment epithelium tear, macular atrophy, and subretinal hemorrhage).

The heterogeneity in response to anti-VEGF drugs or PDT may be explained by the genetic background of each patient and pharmacogenetic characteristics. In this regard, depending on the anti-VEGF drug and/or pathology, many genetic variants, especially single nucleotide polymorphisms (SNP), have been significantly related to variable response to these drugs (Table 1).

Specifically, the *CXCL8-251AA* genotype and minor allele (A) were associated with nonresponse to bevacizumab treatment in European exudative AMD patients (*p* = 0.026) [22]. In the *VEGFA* gene, the *VEGFA-2578C* allele has been related to an increased response to ranibizumab among AMD patients who had not been previously treated (*p* = 0.019) [23]. Carrying the *VEGFA-c.232-28C>T* SNP has been associated with increased response to bevacizumab or ranibizumab in people with macular degeneration, as compared to genotype *CC* after 3, 6, and 12 months of treatment [24], and *VEGFA*- rs833069 *CC+CT* genotypes have been demonstrated to have greater decreases in central subfield macular thickness (CSMT) between baseline and 3 (*p* = 0.002) or 6 (*p* = 0.001) months of treatment, as compared to those with the *TT* genotype [25]. In the *CFH* gene, the *CFH Y402H* SNP was significantly related to higher doses of bevacizumab in AMD patients (*p* = 0.02) [25]. The *TT* genotype for this SNP was associated with a greater improvement in mean visual acuity (*p* = 0.009) in macular degeneration treated with ranibizumab [25], while the *CC* genotype was associated with a decreased response if treated with bevacizumab [26]. Furthermore, the *CFH Y402H TT* genotype was related to a decreased response to PDT in AMD patients (*p* = 0.04) [27]. The *NRP1* rs2070296 *T* allele [28] and *ARMS2* rs10490924 *TT* [29] genotype have been associated with decreased response to bevacizumab and ranibizumab, respectively. Finally, the *HTRA1-625A/G* variant was associated with a decreased response to bevacizumab in people with macular degeneration (*AA* vs. *GG*; *p* = 0.006) [29].

All these drug–gene interactions have been reported as Level 3 evidence; this means they are based on a single significant (not yet replicated) study or annotation, evaluated in multiple studies but lacking clear evidence of an association [21]. As reported in Table 1, anti-VEGF drugs have been related to variable response depending on *CXCL8*, *VEGFA*, *NRP1*, *ARMS2*, *CFH,* and *HTRA1* genotypes in macular degeneration and depending on *VEGFA* genotype in CNV patients, without differentiating results among PCV patients.

These drug–gene interactions have been reported without considering the differences among (non)exudative macular degeneration and CNV or PCV patients, despite pathophysiology and/or therapeutic differences. Moreover, many published manuscripts have studied the association between genetic variants affecting anti-VEGF drug response in exudative AMD patients, including PCV, but without reporting the categorized results. This might explain why many studies regarding how pharmacogenetic (PGx) variants affect anti-VEGF drug response in exudative/wet AMD patients have reported results with moderate/high rates of heterogeneity [30,31,32].

Thus, the aim of this study is to explore the genetic variants affecting anti-VEGF drug response in PCV patients only, considering that physio-pathogenic differences between PCV patients and nAMD patients may explain the heterogeneity in the reported results about genetic variants affecting anti-VEGF drug response.

## 2. Materials and Methods

### 2.1. Search Strategy and Inclusion/Exclusion Criteria

A systematic review was performed. In order to identify all relevant studies about PGx on anti-VEGF drug response in PCV patients, we searched terms in Pubmed on 1 March 2020, using the following argument: (“ranibizumab” OR “bevacizumab” OR “aflibercept” OR “anti-vascular endothelial growth factor” OR “anti-VEGF” OR “choroidal neovascularization” OR “polypoidal choroidal vasculopathy” OR “exudative age-related macular degeneration” OR “neovascular age-related macular degeneration”) AND (“pharmacogenetics” OR “SNP” OR “polymorphism”).

In addition, all the review articles found in the initial search were checked in order to identify the ones containing scientific articles that met the inclusion criteria. Furthermore, we manually checked the provided bibliographies in PharmGKB [21] in order to verify that we included all the relevant manuscripts about genetic variants affecting anti-VEGF drug response.

The obtained scientific articles were included for review, according to the following inclusion/exclusion criteria:

1. Manuscripts studying genetic variants affecting anti-VEGF drug response in oncology patients were excluded.
2. Manuscripts published in journals not indexed in Journal Citation Reports (JCR) were excluded.
3. Manuscripts regarding genetic variants related to the illness and not related to anti-VEGF drug response were excluded.
4. Manuscripts studying the association of genetic variants with response to anti-VEGF drugs in non-PCV patients (only) were excluded.
5. Manuscripts written in English (only) were included.
6. Manuscripts studying the association of genetic variants with patients′ response to anti-VEGF drugs in PCV patients were included.

### 2.2. Data Extraction and Quality Assessment

The search strategy was performed by two independent researchers. Discrepancies were assessed by those two plus three other researchers and finally considered to be included when at least three of them agreed with the inclusion/exclusion decision. A quality assessment of the included analysis was performed using the Newcastle–Ottawa quality assessment scale (NOS) [33]. In this regard, we judged each study on three categories (selection, comparability, and exposure) and eight items, up to nine “stars/points”, as the top score. Finally, those manuscripts with NOS score under five points were excluded from the analysis. We obtained the following information from the included studies: author, treatment strategy, ethnicity, follow-up time, number of patients, gene, SNP, reference SNP (rs), studied endpoint, and minor allele frequency (MAF) and genotype distribution for each of the included SNPs.

Among all the publications found, we included in the meta-analysis those patients with PCV treated with ranibizumab, bevacizumab, or aflibercept, without exclusions regarding grade of severity, stage of progression, comedications, age, sex, or ethnicity conditions. Every genetic variant assessed to be related to anti-VEGF drug response was included, and all the efficacy or toxicity parameters used to evaluate the association between genetic variants and patients′ drug response were recorded and considered for meta-analysis.

### 2.3. Data Analysis

We conducted a random-effects meta-analysis in recessive and dominant models for the T risk allele to investigate the association between the genetic variant *ARMS2 A69S* (rs10490924) and treatment response to anti-VEGF agents in PCV patients, as it was the only genetic variant meeting the meta-analysis criteria. For each primary study, we calculated the effect size as the standardized difference in means between the two groups being compared. A random-effects meta-analysis was chosen due to the variability in methods across the primary studies. Heterogeneity between primary studies was assessed using the I2 statistic [34]. We used R statistics software, version 3.6.2, with the package “meta” to conduct the meta-analysis (https://CRAN.R-project.org/package=meta).

We used Harbord′s test in order to quantitatively assess publication bias, considering *p*-value < 0.1 as significant statistical publication bias.

## 3. Results

**Figure 1:**
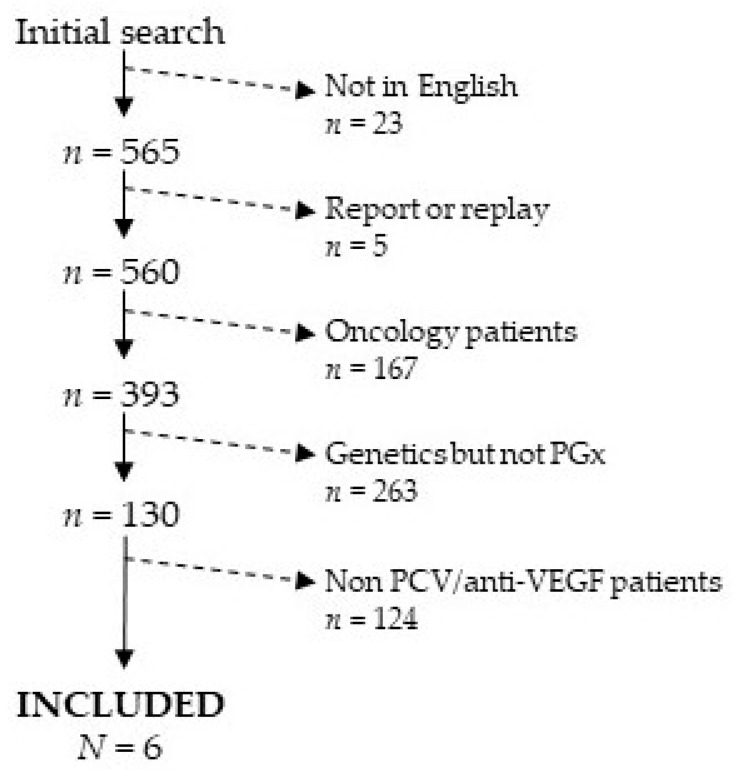
Bibliography search strategy.

In the initial search, we found 588 scientific articles. As defined in exclusion/inclusion criteria, and, after looking through the manuscript′s titles, we excluded 23 articles that were not written in English, five case reports or replies, and 167 articles about anti-VEGF PGx in oncology patients. The remaining manuscript abstracts were checked individually to exclude those dealing with genetic variants related to the illness but not related to drug response (263 manuscripts). Furthermore, in order to differentiate PCV patients among exudative AMD patients, we checked, by hand, the 130 research articles passing the filtering criteria, and we selected only those studying the genetic variants affecting the response to anti-VEGF drugs in PCV patients exclusively (Figure 1).

Finally, six scientific articles were identified as publications exploring genetic variants affecting anti-VEGF drug response in PCV patients [35,36,37,38,39,40]; none of these manuscripts obtained a NOS score under five points, and no evidence of publication bias was found after Harbord′s test. Altogether, these articles investigated 11 different variants in nine genes (Table 2), all MAFs were higher than 0.01, and all of them were in Asian populations (Korea or Japan) treated with anti-VEGF drugs (ranibizumab, bevacizumab, and aflibercept).

In greater detail, three articles included patients treated with PDT in addition to anti-VEGF drugs (36, 39, 40), and three articles included nAMD patients in addition to PCV patients [37,38]. Furthermore, Kawashima Y et al. [37] recruited refractory-to-ranibizumab patients treated with aflibercept and did not provide results that differentiated between PCV and nAMD patients.

As major findings (Table 3), Park UC et al. [35] reported a significant association between *ARMS2 A69S* (rs10490924) and anatomic therapeutic response to ranibizumab or bevacizumab in 81 PCV patients without PDT in a 12-month follow-up. Park DH et al. [36] made the same findings but in 51 patients treated with bevacizumab and PDT, and they also found a significant association with *HTRA1 -625A/G* (rs11200638). Kawashima et al. [37], in AMD (*n* = 15) or PCV (*n* = 26) patients treated with aflibercept, who had been previously treated with ranibizumab, did not report significant results on the relationship between *ARMS2 A69S* (rs10490924), *CFH I62V* (rs800292), or *CFH Y402H* (rs1061170) genetic variants and visual improvement. Hata M. et al. [38], in PCV or AMD patients treated with ranibizumab, did not find any pharmacogenetic association, but, regarding PCV patients only, they found *ARMS2 A69S* (rs10490924) to be related with visual acuity at baseline and 12 months, although not with visual prognosis. Additionally, regarding the second research article by Hata et al. [39], in PCV patients treated only with ranibizumab and PDT, they found the T risk allele in *ARMS2 A69S* (rs10490924) to be related to higher rates of recurrence and a reduction of visual acuity between 12 and 24 months of treatment. Finally, Nakai et al. [40] concluded, among PCV patients, that G allele in *ARMS2 A69S* (rs10490924) is likely associated with a lower chance of retreatment after aflibercept and PDT.

### Meta-Analysis

In the considered publications after systematic review, *ARMS2 A69S* (rs10490924) was the only genetic variant assessed to be related to anti-VEGF drug response in PCV patients in more than two of these publications and the only one related to an endpoint feasible enough to be compared. In this regard, we performed a meta-analysis about the influence of *ARMS2 A69S* on anti-VEGF drug response in PCV patients, considering best-corrected visual acuity (BCVA) improvement as the endpoint. To do this, BCVA data was recorded in baseline and follow-up time for each of the included manuscripts, and these BCVA values were harmonized due to the differences in the used scales. We included in the meta-analysis five out of the six selected publications in the systematic review [35,36,37,39,40]. One of the articles by Hata et al. [38] might not be included because of a lack of information about BCVA values, depending on genotypes and periods.

Finally, about the influence on anti-VEGF drug response, we found that *ARMS2 A69S* (rs10490924) was not statistically significant for both the recessive model (SMD = 0.29; 95% CI = −0.11 to 0.68; *p*-value = 0.28) and the dominant genetic model (SMD = 0.31; 95% CI = −0.14 to 0.75; *p*-value = 0.42). This is probably explained by differences in illness (PCV or AMD), treatments (ranibizumab and/or bevacizumab and/or aflibercept; with/without PDT) and ways of assessment of BCVA.

**Figure 2:**
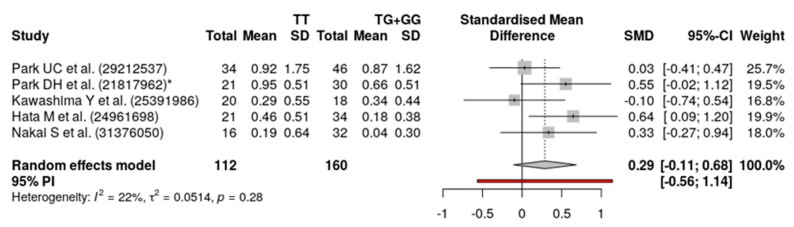
Forest plot of the meta-analysis showing the association between *ARMS2 A69S* (rs10490924) and treatment response to anti-VEGF agents in PCV patients using random-effects models. Recessive model for allele T (TT vs. TG + GG).

**Figure 3:**
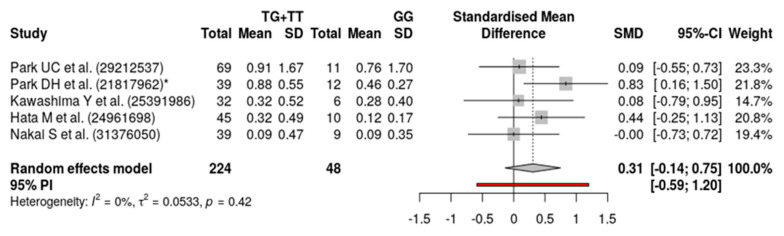
Forest plot of the meta-analysis showing the association between *ARMS2 A69S* (rs10490924) and treatment response to anti-VEGF agents in PCV patients using random-effects models. Dominant model for allele T (TG + TT vs. GG).

As we can see (Figure 2 and Figure 3), our results show a high statistical homogeneity (I^2^ = 0%) in the dominant model and a low statistical heterogeneity (I^2^ < 25%) in the recessive model.

## 4. Discussion

For the first time, we have performed a systematic review of genetic variants affecting anti-VEGF drug response exclusively in PCV patients, considering PCV a separate analysis group that is independent of nAMD.

nAMD differs in the course of the disease and treatment response to PCV. They are phenotypically distinct. In addition, there are single nucleotide polymorphisms (SNPs) in certain genes that determine a greater susceptibility to PCV or nAMD. The G allele of the SNP *162V* (rs8002921) in the *CFH* gene is a risk allele for drusen, more choroidal neovessels, and nAMD. The A allele is a risk allele for thick choroid, choroidal hyperpermeability, less choroidal neovessels, and PCV [41]. *ARMS2 A69S* risk allele frequency was lower in pachychoroidal neovasculopathy but slightly higher in nAMD [42]. In patients treated for nAMD and PCV, after the initial three intravitreal injections, the need for retreatment has been associated with the T (risk) allele of *ARMS2 A69S* and the C (risk) allele of *CFH* rs1329428. The pharmacogenetic study allowed the study of the association of the allelic variant present in the patient and the need for retreatment, and it would be informative for both patients and physicians to predict the number of additional injections [43].

Our literature search has resulted in six studies reporting results on the specific effect of genetic variants in the treatment of PCV. The two most recurrent variants within these studies are *ARMS2 A69S* (rs10490924) and *CFH Y402H* (rs1061170), although other genetic variants such as *HTRA1-625A/G* (rs11200638) and *CFH I62V* (rs800292) have also been related to variable response to anti-VEGF drugs. In any case, results among the different studies are inconclusive. On the other hand, Kawashima et al. [37] and Hata et al. [38] did not find statistically significant results. This may be explained by the small sample size and the inclusion of both PCV and AMD patients altogether. In this study by Hata et al., anti-VEGF drugs seemed not to be useful against nAMD or PCV. Furthermore, in our meta-analysis, we did not observe any statistically significant association between anti-VEGF drug efficacy and the *ARMS2 A69S* variant, despite the influence of the population from the study of Park UC. et al. As we can see in Figure 2, after processing the data for the meta-analysis in order to compare *ARMS2 A69S TT* vs. *TG + GG* patients, data from Park UC do not seem to be statistically significant, which may be explained by the genetic model.

The *HTRA1* gene encodes the expression of heat shock proteins, which are responsible for protection against cell damage due to oxidative stress [44], especially in the retinal pigmented epithelium, photoreceptor, and vascular endothelium. The typical angiogenesis in PCV patients is known to be related to oxidative stress [44], so genetic polymorphisms in *HTRA1* may influence heat shock protein function and response from patients to anti-VEGF drugs.

The *CFH* gene encodes the complement factor H expression, capable of inhibiting several components in the complement cascade that are related to inflammation and drusen appearance and, because of this, with a potential role in the pathogenesis of AMD [45]. Therefore, changes in the coding DNA region of CFH may result in a malfunctioning CFH, rendering it unable to inhibit the complement cascade [46] and, especially, its affinity to C-reactive protein (CRP), enhancing CRP levels in the choroid, which means an enhanced local inflammatory response. All this may, ultimately, lead to increased local levels of VEGF and result in neovascularization [30]. This way, genetic variants in *CFH* may influence anti-VEGF drug response.

Lastly, the *ARMS2* gene encodes a protein, the biological function of which remains unclear [47]. ARMS2 protein is expressed in the choroid but also in monocytes and microglia cells, mediating its activity against apoptotic cells by complement activation [48]. Thus, genetic variants in this gene may lead to decreased apoptotic cell clearance. Irrespective of the exact mechanism, it is reasonable to assume that this variant could influence CNV development [49] and, thus, anti-VEGF drug response.

The main limitation in our meta-analysis is the lack of homogeneity on the clinical criteria defining PCV, AMD, or nAMD patients in studies assessing PCV as an independent analysis group. Actually, in this regard, AMD is divided into three subtypes: nAMD, retinal angiomatous proliferation, and PCV. After the introduction of the pachychoroid concept, PCV is further divided into (A) pachychoroid neovasculopathy, defined as Type 1 neovascularization occurring as secondary to central serous chorioretinopathy or long-standing pachychoroid pigment epitheliopathy without any polyps on indocyanine green angiography (ICGA); (B) PCV, diagnosed on ICGA as the presence of nodular hyperfluorescence, appearing within the first 6 min with/without the presence of an abnormal vascular network. In any case, only this last group of patients was included in our meta-analysis.

The small sample size and an important variability in clinical follow-up evaluation and treatments of PCV/nAMD patients are also limitations to our study. For instance, in their study, Park UC et al. [35] included patients treated with ranibizumab for three months. Meanwhile, a group of patients continued this treatment for six months; others were treated with bevacizumab due to financial reasons. Patients in the study of Park DH et al. received combined therapy of PDT plus intravitreal bevacizumab. Kawashima Y. et al. [37] included patients treated with ranibizumab and switched to aflibercept, regardless of whether they had received previous treatments or not. Hata et al. studied the response to ranibizumab and PDT, while Nakai et al. [40] included patients treated with aflibercept and PDT. Moreover, BCVA measurements (the endpoint variable assessed in the meta-analysis) were taken using different scales, such as Early Treatment for Diabetic Retinopathy Study (ETDRS) and the Landolt or Snellen chart, which hampers the comparison between different studies despite data harmonization. Thus, unifying PCV treatments, scale measurements, and treatment regimens will likely change these results. In addition to these limitations, it is not possible to fully eliminate publication bias, and we may have missed articles in other languages or those rejected for publication because of the absence of significant results.

On the other hand, despite this clinical heterogeneity, our results did not show either statistical or methodological heterogeneity.

Another factor that could have interfered with our results is the small sample size of the studies. Additionally, in two of the six studies (Park UC et al. [35] and Kawashima Y. et al. [37]), researchers exclude analyses from those patients who had to receive PDT as rescue therapy from the pharmacogenetic association. These patients were likely the ones showing the strongest pharmacogenetic effects of the variants; thus, removing them could have altered the pharmacogenetic association analysis results.

Nonetheless, our study also has some strengths. Analyzing PCV patients as an independent analysis group eliminates the bias introduced by the typically larger number of AMD patients, which would have masked the specific pharmacogenetic effects on PCV. Moreover, ethnic differences have been reported regarding the epidemiology of PCV. For instance, PCV is more prevalent in Asians than in Caucasians, and it is also more prevalent in Asian men and Caucasian women. In this sense, our study is multi-institutional, but all the patients included have Asian origin, thus reducing population heterogeneity.

In short, anti-VEGF drugs have shown variable rates of efficacy without considering genetic polymorphisms depending on the patient diagnosis: PCV, nAMD, and even oncological patients. Many genetic polymorphisms have been related to variable response to anti-VEGF drugs, although without considering differences between patient diagnosis and/or concomitant treatment with PDT, with small sample sizes and/or different follow-up times, and without considering the combined effect of different genetic variants related to drug response.

## 5. Conclusions

The genetic polymorphisms *HTRA1 -625A/G* (rs11200638), *CFH I62V* (rs800292), *CFH Y402H* (rs1061170), and, especially, *ARMS2 A69S* (rs10490924) have been related to variable response to anti-VEGF drugs in PCV patients; however, there is a lack of information about the efficacy of anti-VEGF drugs, depending on PCV or nAMD patients, concomitant PDT, and genetic variants affecting these drug responses. Therefore, further studies are needed, considering differences in illness (PCV or AMD), treatments (ranibizumab and/or bevacizumab and/or aflibercept; with/without PDT), follow-up time, genetic models, and considering the combined effects of different genetic variants in a bigger sample size.

## Figures and Tables

**Table 1:** Genetic variants affecting drug response in macular degeneration or choroidal vasculopathy (obtained from PharmGKB [21]).

Ref SNP (rs)	Gene	Drug	Type	Pathology
rs4073	*CXCL8*	bevacizumab	Efficacy	Macular Degeneration
rs699947	*VEGFA*	ranibizumab	Efficacy	Macular Degeneration
rs3025000	*VEGFA*	bevacizumab, ranibizumab	Efficacy	Macular Degeneration
rs2070296	*NRP1*	ranibizumab	Efficacy	Macular Degeneration
rs10490924	*ARMS2*	bevacizumab	Efficacy	Macular Degeneration
rs1061170	*CFH*	bevacizumab	Dosage	Macular Degeneration
rs1061170	*CFH*	bevacizumab, ranibizumab	Efficacy	Macular Degeneration
rs833069	*VEGFA*	ranibizumab	Efficacy	Macular Degeneration
rs11200638	*HTRA1*	bevacizumab, ranibizumab	Efficacy	Macular Degeneration
rs1061170	*CFH*	photodynamic therapy	Efficacy	Macular Degeneration
rs5985	*F13A1*	photodynamic therapy	Efficacy	Choroidal Neovascularization
rs2010963	*VGFA*	bevacizumab, pegaptanib, ranibizumab	Efficacy	Choroidal Neovascularization

**Table 2:** Genetic variants and population characteristics of included manuscripts.

Author	Ref SNP (rs)	Gene	SNP (Location)	MAF	Genotype mm/Mm/MM	Origin	Treatment	Patients	Follow-Up (Months)
Park UC et al. [35]	rs800292	*CFH*	*I62V*	0.290	4/46/45	Korea	Ranibizumab or bevacizumab	PCV	12
	rs1061170	*CFH*	*Y402H*	0.080	1/13/81	Korea	Ranibizumab or bevacizumab	PCV	12
	rs9332739	*C2*	*E318D*	0.019	1/1/93	Korea	Ranibizumab or bevacizumab	PCV	12
	rs641153	*CFB*	*R32Q*	0.081	0/14/79	Korea	Ranibizumab or bevacizumab	PCV	12
	rs429608	*SKIV2L*	*3493G/A*	0.088	1/13/80	Korea	Ranibizumab or bevacizumab	PCV	12
	rs699947	*VEGFA*	*C-2578A*	0.281	10/39/45	Korea	Ranibizumab or bevacizumab	PCV	12
	rs3025039	*VEGFA*	*C936T*	0.247	6/36/53	Korea	Ranibizumab or bevacizumab	PCV	12
	rs10490924	*ARMS2*	*A69S*	0.375	12/40/42	Korea	Ranibizumab or bevacizumab	PCV	12
	rs11200638	*HTRA1*	*-625A/G*	0.370	12/41/42	Korea	Ranibizumab or bevacizumab	PCV	12
	rs1136287	*PEDF*	*Met72Thr*	0.488	23/50/22	Korea	Ranibizumab or bevacizumab	PCV	12
Park DH et al. [36]	rs10490924	*ARMS2*	*A69S*	0.412	12/18/21	Korea	Bevacizumab + PDT	PCV	12
	rs11200638	*HTRA1*	*-625A/G*	0.382	10/19/22	Korea	Bevacizumab + PDT	PCV	12
Kawashima Y et al. [37]	rs10490924	*ARMS2*	*A69S*	0.316	6/12/20	Japan	1st Ranibizumab; 2nd Aflibercept	PCV or nAMD	6
	rs800292	*CFH*	*I62V*	0.184	2/10/26	Japan	1st Ranibizumab; 2nd Aflibercept	PCV or nAMD	6
	rs1061170	*CFH*	*Y402H*	0.145	1/9/28	Japan	1st Ranibizumab; 2nd Aflibercept	PCV or nAMD	6
Hata M et al. [38]	rs10490924	*ARMS2*	*A69S*	0.400	17/43/45	Japan	Ranibizumab	PCV or nAMD	24
	rs800292	*CFH*	*I62V*	0.246	7/38/61	Japan	Ranibizumab	PCV or nAMD	24
Hata M et al. [39]	rs10490924	*ARMS2*	*A69S*	0.338	10/32/35	Japan	Ranibizumab + PDT	PCV	24
	rs800292	*CFH*	*I62V*	0.316	10/28/38	Japan	Ranibizumab + PDT	PCV	24
Nakai S et al. [40]	rs10490924	*ARMS2*	*A69S*	0.427	9/23/16	Japan	Aflibercept + PDT	PCV	12

Ref SNP (rs): reference single nucleotide polymorphism; MAF: minor allele frequency; mm: number of patients with recessive homozygous genotype; Mm: heterozygous genotype; MM: dominant homozygous genotype, PDT: photodynamic therapy; PCV: polypoidal choroidal vasculopathy; nAMD: neovascular age-related macular degeneration.

**Table 3:** Genetic variants and related endpoints of included manuscripts.

Study	*n*	Gene	Change	Related Endpoint	*p*-Value
Park UC et al. * [35]	81	*CFH*	*I62V*	BCVA (Early Treatment Diabetic Retinopathy Study)	0.039
				Total Foveal Thickness change	0.255
				Pigment Epithelium Detachment (PED) regression on OCT	0.079
	81	*CFH*	*Y402H*	BCVA (Early Treatment Diabetic Retinopathy Study)	0.043
				Total Foveal Thickness change	0.551
				Pigment Epithelium Detachment (PED) regression on OCT	0.133
	80	*ARMS2*	*A69S*	BCVA (Early Treatment Diabetic Retinopathy Study)	0.338
				Total Foveal Thickness change	0.212
				Pigment Epithelium Detachment (PED) regression on OCT	0.004
	81	*HTRA1*	*-625A/G*	BCVA (Early Treatment Diabetic Retinopathy Study)	0.615
				Total Foveal Thickness change	0.276
				Pigment Epithelium Detachment (PED) regression on OCT	0.014
Park DH et al. [36]	51	*ARMS2*	*A69S*	FA-GLD	0.004
				ICGA-GLD	0.972
				Complete absence of leakage by FA	0.04
				Complete polyp regression by ICGA	0.006
				BCVA (Snellen visual acuity)	0.034
	51	*HTRA1*	*-625A/G*	FA-GLD	0.009
				ICGA-GLD	0.937
				Complete absence of leakage by FA	0.019
				Complete polyp regression by ICGA	0.002
				BCVA (Snellen visual acuity)	0.022
Kawashima Y et al. **^†^** [37]	38	*ARMS2*	*A69S*	Visual acuity change (Landolt chart)	0.91
	38	*CFH*	*I62V*	Visual acuity change (Landolt chart)	0.44
	38	*CFH*	*Y402H*	Visual acuity change (Landolt chart)	0.24
Hata M et al. [38]	70	*ARMS2*	*A69S*	BCVA (Landolt chart)	0.942
	70	*CFH*	*I62V*	BCVA (Landolt chart)	0.352
Hata M et al. [39]	77	*ARMS2*	*A69S*	BCVA (Landolt chart) at 12 months/24 months	0.957/0.048
	76	*CFH*	*I62V*	BCVA (Landolt chart) at 12 months/24 months	0.439/0.664
Nakai S et al. [40]	48	*ARMS2*	*A69S*	BCVA	0.235
				Central Retinal Thickness	0.381
				Subfoveal Choroidal thickness	0.133

* Only genetic variants and parameters included in at least one of the other chosen publications are shown. ^†^ *p*-Value is referred to nAMD patients without distinguishing PCV patients. BCVA: best corrected visual acuity; OCT: optical coherence tomography; FA: fluorescein angiography; GLD: greatest lineal dimension; ICGA: indocyanine green angiography.
